# Growth in spending for substance abuse treatment services for the privately insured population

**DOI:** 10.1186/1940-0640-10-S1-A66

**Published:** 2015-02-20

**Authors:** Cindy Parks Thomas, Tami L Mark, Dominic Hodgkin, Katharine Levit

**Affiliations:** 1 Schneider Institutes for Health Policy, Brandeis University, 415 South Street MS035, Waltham MA, 02454, USA; 2 Truven Health Analytics, 7700 Old Georgetown Road, Bethesda, MD, 20814, USA

## Background

Through 2009, private-sector spending on substance use disorder (SUD) treatment services has been a small and stable portion of commercial health-care spending (0.4% in 2003 to 2009). This study examines growth in spending for SUD treatment services extended through 2012 among the privately insured, to assess changing patterns in light of parity legislation, changes in the economy, and availability of new treatments.

## Materials and methods

Spending data for 2004–2012 were compiled from the Truven Health MarketScan® commercial database, which contains private insurance claims for large employers, representing 66 million covered lives nationally. Per enrollee, all health and SUD treatment utilization and spending were calculated for all services, inpatient care, outpatient care, and prescription drugs.

## Results

Between 2004 and 2012, per capita spending on SUD treatment services for the commercially insured increased by 15.4 percent annually, compared to 4.4 percent for all services, and 5.4 percent for mental health services. After 2009, overall health spending growth decreased from 5.2 percent to 3.1 percent annually, while SUD spending rapidly escalated, more than doubling (10.4% to 24.2% annually). This acceleration occurred for inpatient and outpatient care.

Outpatient care, particularly for drug abuse services, accounted for two-thirds of 2009–2012 spending growth, increasing at 33.8 percent annually. Price-per-outpatient visit grew at 2.3 percent prior to 2009, and 14 percent after. Outpatient visit utilization growth tripled (from 5.1% to 16.6% annually). While prior to 2009, SUD treatment medications grew rapidly (from a small base), spending growth slowed after 2009 (especially alcohol treatment medications), similar to that of general health medications.

## Conclusions

While a small part of all health spending, private-sector SUD spending on nonprescription care has continued to accelerate compared to spending on all health, particularly for the outpatient sector, and particularly after 2009. While it is not clear the exact causes of this increase, some potential factors occurring concurrently include: implementation of parity laws at the Federal level and in more states, and the economic downturn, which may have affected use of SUD services differently than general health services.

